# The Impact of Fucoidan Extracts on Heat-Stress-Induced Loss of In Vitro Fast-Twitch Muscle Function in Mice

**DOI:** 10.3390/muscles4010006

**Published:** 2025-02-27

**Authors:** Samantha T. C. Kucewicz, Stefan Piantella, Jarrod E. Church, Caroline J. Taylor, Chris van der Poel

**Affiliations:** 1 Department of Microbiology, Anatomy, Physiology & Pharmacology, School of Life Sciences, La Trobe University, Bundoora, VIC 3086, Australia; s.kucewicz@latrobe.edu.au (S.T.C.K.); j.church@latrobe.edu.au (J.E.C.); c.taylor@latrobe.edu.au (C.J.T.); 2 Department of Psychology, Counselling & Therapy, School of Psychology and Public Health, La Trobe University, Bundoora, VIC 3086, Australia; s.piantella@latrobe.edu.au

**Keywords:** skeletal muscle contraction, heat stress, fucoidan, Fucus vesiculosus, Undaria pinnatifida

## Abstract

Elevated temperatures have been shown to decrease muscle force production, with potential causes including protein unfolding, enzyme denaturation, and reactive oxygen species (ROS). This study aimed to investigate whether fucoidan, a compound derived from brown seaweed, could mitigate heat-stress-induced loss of muscle function. C57BL/6 mice were orally administered fucoidan (400 mg/kg/day) from one of two different seaweed species Fucus vesiculosus (FVF) or Undaria pinnatifida (UPF) or vehicle control for seven consecutive days. Subsequently, the in vitro muscle function of the fast-twitch extensor digitorum longus (EDL) was assessed at either 25 °C (control) or 43 °C (heat stress). Functional analysis was complemented with gene analysis and the C2C12 myoblast heat-stress assay. The temperature (43 °C)-induced loss of force produced by the EDL muscle was significantly attenuated by fucoidan from FVF but not UPF. Fucoidan from UPF did not affect gene expression levels, whereas fucoidan from FVF significantly increased the expression levels of HSP90. In mouse C2C12 myoblasts, heat stress induced a significant increase in ROS production which was significantly reduced by both fucoidan species. These results suggest fucoidan extracted from Fucus vesiculosus may be an effective preventive strategy to protect against heat-induced loss of muscle strength in fast-twitch muscles.

## 1. Introduction

Mammalian skeletal muscle force production, along with contraction and relaxation rates, is significantly influenced by temperature. Experiments using isolated skeletal muscle from rodents have demonstrated that, at temperatures below 35 °C, there is a marked decrease in both maximum tension and the rate of force production [1,2,3]. Contractile properties exhibit minimal changes between 35–40 °C, with only slight increases in contraction rates and force production [4]. As temperatures are increased above 35 °C towards the body core temperature (37 °C), there are marked increases in the rate of muscle contraction and the amount of force produced [5,6]. These effects are largely fiber-type-dependent, with fast-twitch fibers being more sensitive to high temperatures than slow-twitch muscles. In fact, tetanic contractions and fatigue of the isolated slow-twitch soleus muscle are almost unaffected at temperatures between 37 °C and 43 °C [7].

In human participants, the effect of temperature on skeletal muscle function is less equivocal. Below muscle temperatures of 33 °C, there is a reduction in maximal voluntary contraction force, as well as a reduced speed of contraction [8,9,10]. The maximal force and peak power produced during a cycle test have been shown to be significantly higher at muscle temperatures of 39 °C compared to 36 °C [11]. However, muscular strength and endurance were decreased following a 30 min 65–75 °C sauna exposure [12]. De Ruiter and de Haan [13] observed no change in tetanic force when there was an increase in calculated muscle temperatures between 31 °C and 39 °C. However, there have been significant increases in the rate of force development and force relaxation [13,14], with no temperature-induced changes in muscle EMG activity [15], suggesting temperature effects are mediated through the intracellular Ca^2+^-handling properties of skeletal muscle cells. Temperatures above 40 °C is a significant stressor which can have detrimental effects on human health [16], including the impairment of skeletal muscle function [17], and alterations in muscle metabolism [18]. Studies using isolated rodent muscle preparations have shown that, at temperatures above 43 °C, there is a greater than 60% decrease in force production within 20–30 min at elevated temperatures [19]. While the precise cause of the loss of force production at temperatures above 40 °C is not known, the production of reactive oxygen species (ROS) at elevated muscle temperatures has been hypothesized as a cause.

Reactive oxygen species are produced during repeated contractions such as those resulting in muscle fatigue [20]. In rat diaphragm muscles, it has been shown that there is an increase in ROS production, in particular, superoxide (O_2^−^_), during fatigue-inducing contractions [21,22]. The increase in ROS production seen with fatiguing contractions is known to be inversely related to the magnitude of fatigue observed [23]. Importantly, at muscle temperatures above 42 °C, there is a significant increase in O_2^−^_ production that is prevented by the addition of the O_2^−^_ scavenger, Tiron [24]. When Tiron was added to isolated muscle bundles from flexor digitorum brevis muscles, the muscles were more resistant to the temperature-associated increase in muscle fatigue [25], lending strength to the hypothesis that ROS production plays an important role in the effect of temperature on muscle contraction. While the role of oxidative stress and antioxidant supplementation in sports performance and training adaptation remains equivocal [26], only one study has investigated any protective effect that antioxidants may have on temperature-induced changes to muscle function. Diaz and colleagues [27] investigated the potential use of the strong antioxidant N-acetylcysteine (NAC) in reducing or preventing the temperature-induced loss of muscle function. The aforementioned study demonstrated that isolated rat diaphragm strips fatigue faster at 37 °C compared to 22 °C and, when NAC was present, temperature had less of an influence on the rate of fatigue. More recently, NAC was shown to increase antioxidant and heat shock protein levels and alleviate oxidative stress in heat-stressed skeletal muscle cells [28]. While it is yet to be investigated as a potential treatment in reducing heat stress in human patients, taken together, these results support the investigation of other antioxidants as a potential strategy to mitigate the harmful effects of free radicals generated at muscle temperatures above 40 °C.

Fucoidan is a sulfated polysaccharide found in the cell wall of various species of brown seaweed. Fucoidan has garnered considerable attention due to its wide range of biological activities, including but not limited to anti-inflammatory, anti-viral, anti-thrombotic, and antioxidant properties [29,30]. While fucoidan has been shown to possess a high antioxidant capacity [31,32,33,34,35], the free radical scavenging ability is heavily influenced by the species of origin, its structure, and the extraction method [36]. To further confound the biological properties of fucoidan, cell type was recently identified as a factor influencing the antioxidant capacity of fucoidan, as fucoidan-mediated protection against oxidative stress was found to occur in retinal but not in neuronal cells [37]. Importantly, the antioxidant effects of fucoidan in skeletal muscle cells are to be determined, and the potential effects of fucoidan on heat-stress-induced muscle dysfunction remain unexplored. In this study, we aimed to investigate the protective effects of fucoidan extracted from two brown seaweed species, Fucus vesiculosus (FVF) and Undaria pinnatifida (UPF), against heat-stress-induced loss of muscle function.

## 2. Results

### 2.1. Heat-Stress-Induced Loss of EDL Function

**Figure 1:**
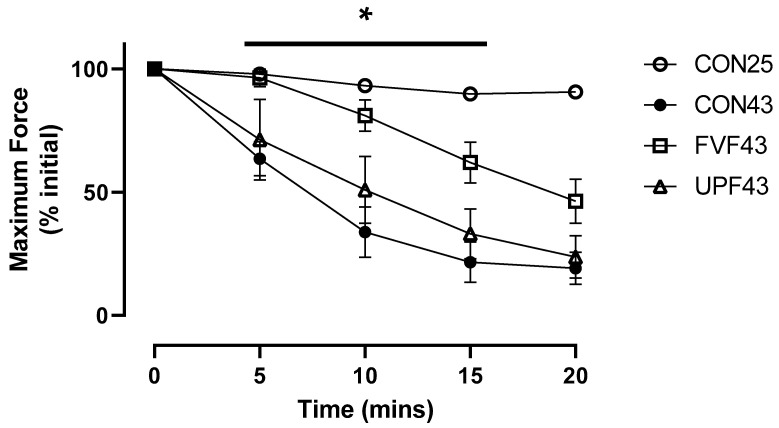
Maximum tetanic force decline (% initial) in mouse fast-twitch EDL muscles at 25 °C (N = 5, open circles) and 43 °C (n = 5, solid circles) for 20 min. Maximum tetanic force declined at 43 °C in mouse fast-twitch EDL muscles from mice that received 7 consecutive days of supplementation with Fucus vesiculosus (FVF43, N = 5, open square) or Undaria pinnatifida (UPF43, N = 7, open triangle). Data are presented as mean ± SEM with level of significance set at *p* < 0.05. Significant difference between FVF43 and CON43 shown by * as determined by 2-way ANOVA, Šidāk multiple-comparisons analysis.

Consistent with previous studies, there was a significant elevated temperature effect on force production that was time-dependent [4,5,17,19,38]. When comparing the force responses for muscles at room temperature (CON25) with muscles at 43 °C (CON43), there was a main effect for both temperature [F (1, 8) = 53.45, *p* < 0.0001] and time [F (1.1, 9.4) = 90.65, *p* < 0.0001], as well as a significant temperature–time interaction [F (4, 32) = 54.45, *p* < 0.0001] (Figure 1). At all time points following the 15 min incubation period (5, 10, 15, and 20 min), maximum tetanic force production at 43 °C (CON43) (mean ± SEM, 63.53 ± 6.97%, 33.73 ± 10.24%, 21.52 ± 8.22%, and 19.13 ± 6.52%, respectively) was significantly lower (*p* < 0.05, two-way ANOVA Šidāk post hoc test) than the stable force production developed at 25 °C (CON25) (97.87 ± 0.56%, 93.17 ± 1.47%, 89.76 ± 1.51%, and 90.63 ± 1.31%, respectively) (Figure 1).

When comparing the force responses of EDL function in the experimental groups at 43 °C (CON43, FVF43, and UPF43), there was a significant time [F (1.9, 27.4) = 99.10, *p* < 0.0001], treatment [F (2, 14) = 5.31, *p* < 0.05], and interaction [time x treatment, [F (8, 56) = 3.55, *p* < 0.05] effect (Figure 1). The Šidāk multiple-comparisons post hoc analysis showed that EDL muscles isolated from mice treated for one week with UPF (UPF43) had a decline in maximum force production at 43 °C that was not significantly different from the force production of heat-stress control (CON43) mice (Figure 1, *p* > 0.05). This result demonstrates that UPF did not impact the heat-stress-induced loss of force production. However, the force productions at 5, 10, and 15 min into the heat-stress protocol were significantly higher in the FVF43 group (96.28 ± 3.58, 81.08 ± 6.38, and 61.96 ± 8.28%, respectively) compared to both the CON43 (63.53 ± 6.97%, 33.73 ± 10.24%, and 21.52 ± 8.22%, respectively) and UPF43 groups (71.24 ± 16.28, 50.85 ± 13.57, and 33.08 ± 10.11%, respectively) (*p* < 0.05, Šidāk multiple-comparisons). This suggests that the FVF treatment was able to slow the impact of elevated temperature on EDL force production. FVF did not prevent the loss of force production as seen at 20 min into the heat-stress protocol, where the force produced by FVF43-treated mouse EDL muscles was not significantly different to the forces produced in the CON43 and UPF43 experimental groups.

### 2.2. Heat Shock Protein Expression Following Heat Stress

**Figure 2:**
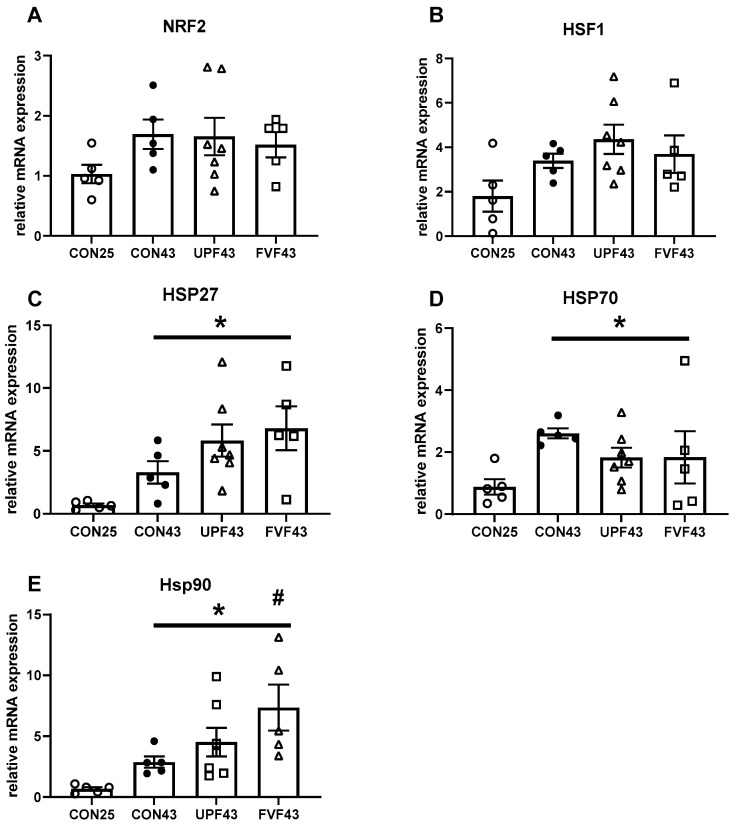
Effects of temperature and fucoidan species on mRNA expression in EDL muscles. Markers of oxidative-stress response (**A**,**B**), and heat-stress response (**C**–**E**). * *p* < 0.05 compared to CON25, # *p* < 0.05 compared to CON43; 1-way ANOVA with Šidāk multiple-comparisons analysis. N = 5 for CON25 (open circle), N = 5 for CON43 (closed circle), N = 7 for Undaria pinnatifida (UPF43, closed triangle), and N = 5 for Fucus vesiculosus (FVF43, open triangle). Data are presented as mean ± SEM.

When comparing transcription levels of genes known to be influenced by heat stress, we did not observe a statistical significant increase in either the expression of the transcription factors NRF2 (CON25: 1.03 ± 0.15 compared to CON43: 1.69 ± 0.24, t (8) = 2.29, *p* = 0.051) or HSF1 (CON25: 1.80 ± 0.69 compared to CON43: 3.4 ± 0.32, t (8) = 2.07, *p* = 0.073) (Figure 2A,B). In contrast, after exposure to 43 °C (CON43), there was a significant increase in the gene expression levels of all heat shock proteins measured, HSP27 (t (8) = 2.91, *p* = 0.019) and HSP70 (t (8) = 5.82, *p* = 0.0004) and HSP90 (t (8) = 4.48, *p* = 0.002), compared to the CON25 group (0.68 ± 0.13, 0.88 ± 0.25, 0.67 ± 0.14 respectively) (Figure 2C–E).

When comparing the effect of fucoidan on expression levels following heat stress, there was no significant treatment effect with a significant difference between CON43, UPF43, or FVF43 gene expression levels of NRF2 (F (2, 14) = 0.09, *p* = 0.91), HSF1 (F (2, 14) = 0.61, *p* = 0.55), HSP27 (F (2, 14) = 1.61, *p* = 0.23), or HSP70 (F (2, 14) = 0.78, *p* = 0.48). Interestingly, FVF, but not UPF, significantly increased the expression levels of HSP90 following 43 °C compared to control [FVF43 compared to CON43, F (2, 14) = 5.1, *p* = 0.016 (Figure 2E)].

### 2.3. Heat-Induced ROS Production in C2C12 Myoblasts

**Figure 3:**
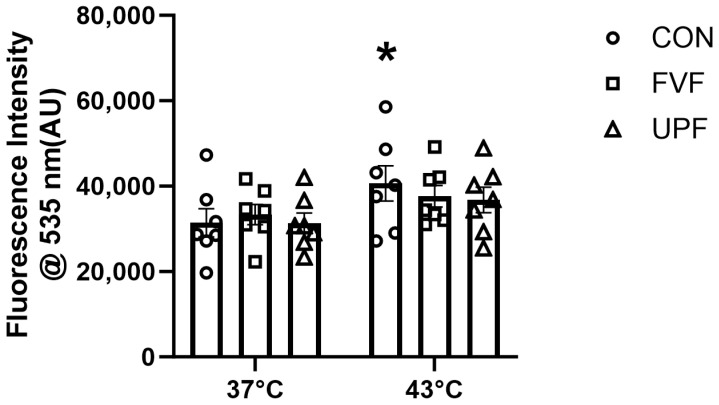
Reactive oxygen species (ROS) production as measured by fluorescence intensity of C2C12 cells at 37 °C and following 2 h of 43 °C heat-stress treatment. Temperature was found to significantly impact C2C12 cell ROS production in the vehicle control group (CON, N = 7, open circle). There was no statistically significant difference between ROS production at 37 °C and 43 °C in either the Fucus vesiculosus (FVF, N = 7, open square) or Undaria pinnatifida (UPF, N = 7, open triangle) groups. Data are presented as mean ± SEM with level of significance set at *p* < 0.05 (* represents significant difference between CON 37 °C and CON 43 °C).

ROS production in C2C12 cells was measured through fluorescent intensity resulting from the ROS oxidation of the H2DCFDA oxidation of DHE. Consistent with previously published heat-stress results in C2C12 cells that have shown an increase in ROS production at 43 °C [39,40], we observed a significant increase in fluorescence intensity at 43 °C (40,635 ± 4134 AU) compared with the control 37 °C (31,408 ± 3280 AU) (main effect for temperature, F (1, 36) = 6.61, *p* < 0.05, Figure 3). When C2C12 cells were exposed to 43 °C in the presence of fucoidan species, there was no significant main effect for fucoidan treatment on ROS production [F (2, 36) = 0.23, *p* = 0.8]. The Šidāk multiple-comparisons test confirmed the ability of both fucoidan species to prevent ROS production at 43 °C, as there was no statistically significant difference between 37 °C and 43 °C fucoidan-treated experimental groups (UPF; 31,319 ± 2348 and 36,754 ± 2998 AU, respectively; and FVF; 33,320 ± 2386 and 37,644 ± 2542 AU, respectively) (Figure 3). This supports our hypothesis that fucoidan has antioxidant potential skeletal muscle C2C12 cells.

## 3. Discussion

In this study, we investigated the effect of fucoidan extracted from two different brown seaweed species, Undaria pinnatifida and Fucus vesiculosus, on the heat-stress-induced loss of skeletal muscle force production. Our results indicate that, while UPF had no significant effects on the loss of force, FVF significantly attenuated the heat-induced force loss at 5, 10, and 15 min. Additionally, our findings suggest that the mechanism behind the protective effect of FVF may involve the modulation of reactive oxygen species (ROS) production and the upregulation of heat shock protein 90 (HSP90) expression. These results provide insights into the potential use of fucoidan, extracted from Fucus vesiculosus, as a protective agent against heat-stress-induced skeletal muscle dysfunction.

The effect of temperature on skeletal muscle function has been extensively studied, showing that temperatures above 40 °C cause a significant decrease in isolated skeletal muscle force production [4,5,17,41,42,43]. Importantly, in the present study, the results support this outcome of temperatures above 40 °C causing a significant loss of fast-twitch EDL muscle force production (Figure 1). Although the effect of temperature on muscle function has been extensively studied, the mechanism responsible for the temperature induced loss of muscle function is largely unknown. However, modulating ROS production has been a popular target to combat heat stress with many promising strategies containing antioxidant properties [44,45,46,47]. The antioxidant capacity of fucoidan isolated from various brown seaweed species has been repeatedly demonstrated [48,49,50] and was the primary reason why fucoidan was identified as a potential strategy to attenuate the impairment of muscle function at elevated temperatures. In the present study, we provide further evidence for the potent antioxidant properties of fucoidan, specifically in a skeletal muscle cell culture model of heat stress and show that fucoidans extracted from both Undaria pinnatifida and Fucus vesiculosus decreased heat-stress-induced ROS production to levels equivalent to control levels.

In the current study, we demonstrated that the temperature-induced loss of force production was reduced but not prevented by a one-week oral supplementation with fucoidan from Fucus vesiculosus. Interestingly, the temperature-induced loss of skeletal muscle force production was not influenced by fucoidan from Undaria pinnatifida. It is well-established that the bioactive properties of fucoidan are dependent on the extraction techniques, brown algae species of origin, season of harvest, geographical location, algal maturity, and which component of the brown algae the fucoidan was extracted from [51]. The fucoidan used in this study that was extracted from Fucus vesiculosus had a higher yield of fucose and polyphenols compared to Undaria pinnatifida (Table 1). Fucose is the main component of fucoidan; higher levels of fucose and polyphenols indicate a purer compound and higher antioxidant properties [52]. Another important consideration for the biological activity of fucoidans is the molecular weight of fucoidan. Heavily influenced by extraction methods and species of origin, the molecular weight of fucoidan typically ranges between 13 to 1500 kDa with the classification of fucoidans being divided into low-molecular-weight fucoidan (LMWF < 10 kDa), medium-molecular-weight fucoidan (MMWF 10–1000), and high-molecular-weight fucoidan (HMWF > 1000 kDa) [53]. The importance of molecular weight on biological activity has previously been demonstrated in vitro, where MMWF inhibited angiogenesis and LMWF promoted angiogenesis in human umbilical vein endothelial cells [54]. The molecular weight of fucoidan has been shown to impact the pharmacokinetics and tissue distribution of fucoidan. Previous studies have shown that doses as low as 30 mg/kg of LMWF (9.6 kDa) result in a plasma mean residence time (MRT) of 9.7 h [55] compared to a MRT of 6.7 h following the ingestion of 100 mg/kg of an MMWF (935 kDa) [56]. In this study, both FVF and UPF species reduced ROS production in C2C12 cells, while having different biological effects on the heat-stress-induced loss of EDL muscle function. Future studies should determine whether fucoidans of lower molecular weight and higher MRT have the same or more beneficial effects on skeletal muscle and whether there are any fiber-type-dependent differences in the fucoidan distribution and effect. Despite different effects on the temperature-induced loss of EDL force production, fucoidan from both species reduced ROS production in C2C12 cells, suggesting oxidative stress may not be the predominant cause of the heat-stress-induced loss of muscle function as initially hypothesized.

Heat therapy has been shown to promote the expression of factors including VEGF, ANGPT1, ANGPT2, CCL2, and HSPs, all of which are associated with angiogenesis in human skeletal muscle [57]. Our gene expression analysis showed a temperature-induced increase in the gene transcription of all HSP genes analyzed. However, only HSP90 was influenced by fucoidan treatment. Fucoidan from Fucus vesiculosus resulted in an increase in HSP90 levels compared to the control heat-stress group. However, similar HSP90 levels were detected in both the control and Undaria pinnatifida heat-stress-treated groups. HSP90 is a molecular chaperone in most mammalian cells that is involved in protection against cellular stress [58]. In skeletal muscle, HSP90 has been shown to play important roles in cell proliferation and differentiation [59], and sarcomere assembly [60], and has recently been identified as a potential myokine, mediating the exercise-induced immune response [61]. HSP90 has also been shown to provide stability for sirtuin 1 (SIRT1) [62], which is a known regulator of the cellular response to stress by regulating proteins such as p53 and Forkhead box family (FOXO) [63]. Fucoidan from Fucus vesiculosus increases the SIRT1 expression in human epithelial (MiaPaCa-2) cells [64]. Additionally, an important functional relationship between SIRT1 and HSP90 has been observed in B-cell lymphoma (DLBCL) cells, where SIRT1 inhibition reduced heat-stress-induced HSP90 induction [65]. This relationship has also been demonstrated in COS-7 and HepG2 cell lines where HSP90 inhibition reduced the levels of SIRT1 [62]. Recently, it was demonstrated that a single dose of fucoidan (300 mg/kg) reduced angiotensin II-induced cardiac dysfunction in mice through the stabilization and upregulation of SIRT1, a reduction in p53 expression levels, and an increase in Bcl-2 expression levels [66]. Based on these findings, we hypothesize that supplementation with fucoidan from Fucus vesiculosus increases the SIRT1 and HSP90 activity in skeletal muscles, facilitating the maintenance of mitochondrial function and Ca^2+^ homeostasis, subsequently mitigating the loss of muscle force production induced by elevated temperatures.

The temperature-induced loss of muscle function can occur in various situations, often related to extreme environmental conditions or strenuous physical activity such as those performed by soldiers, athletes, firefighters, and outdoor workers. In humans, body temperatures above 40 °C are life-threatening and characterized by multi-organ dysfunction [67]. Given that temperatures of contracting muscles can be up to 1 °C higher than the body core temperature [68], it is no surprise that muscle fatigue, muscle injury, and chronic cramps are among the early symptoms of someone experiencing exertional heat stroke [69,70]. Therefore, identifying preventive strategies, such as fucoidan, that reduce the impact of an elevated temperature on skeletal muscle function could reduce the incidence and progression of exertional heat stroke.

## 4. Materials and Methods

### 4.1. Animals

All procedures involving animals performed in this study were approved by the La Trobe University Animal Ethics Committee (AEC 18068) and conducted in accordance with the Australian Code of Practice for the Care and Use of Animals for Scientific Purposes. Mice (male, C57BL/6) were purchased at 12 weeks of age from the Walter and Eliza Institute of Medical Research (WEHI, Parkville, VIC, Australia), and were housed in standard laboratory conditions of 22 ± 2 °C with a relative humidity of 55 ± 8% and a 12 h light/dark cycle. Mice had access to water and standard chow diet ad libitum.

### 4.2. C57BL/6 Mouse Treatment with Fucoidan

Mice were randomly allocated to a treatment group, Group 1—vehicle control 25 °C (CON25, N = 5), Group 2—vehicle control 43 °C (CON43, N = 5), Group 3—fucoidan extracted from Fucus vesiculosus 43 °C (FVF43, N = 5), or Group 4—fucoidan extracted from Undaria pinnatifida fucoidan 43 °C (UPF43, N = 7). Previous studies have shown that a daily fucoidan dose of 300–620 mg kg.day^−1^ has beneficial effects on skeletal muscle function [71,72], and a daily dose of between 100–400 mg.kg.day^−1^ has been shown to reduce inflammatory pathology associated with colitis [73,74]. Mice in groups 3 and 4 were administered daily doses of fucoidan (400 mg.kg.day^−1^) dissolved in injectable water via oral gavage for 7 consecutive days. Control mice (Groups 1 and 2) were administered equal volumes of injectable water via oral gavage for 7 consecutive days. Fucoidan extracts were produced and provided by Marinova Pty Ltd. (Tasmania, Australia). The chemical composition of the fucoidan species used in this study are described in Table 1 and has been previously reported [72,75].

### 4.3. In Vitro Muscle Function Testing

With the consideration that fast-twitch muscles are more susceptible to elevated-temperature-induced loss of force production, the isometric contractile properties of isolated fast-twitch extensor digitorum longus (EDL) were evaluated in vitro following one week of daily oral gavage of fucoidan or vehicle control, as previously described [76,77,78]. Mice were anaesthetized with sodium pentobarbitone (6 mg/kg), and, once the pedal reflex was unresponsive [79], EDL muscles were surgically removed and transferred to a 1300A 3-in-1 whole Mouse Test System (Aurora Scientific, Toronto, ON, Canada). Krebs Ringer solution (137 mM NaCl, 24 mM NaHCO_3_, 11 mM D-glucose, 5 mM KCl, 1 mM NaH_2_PO_4_H_2_O, and 1 mM MgSO_4_ in ultrapure water), constantly bubbled with carbogen (5% CO_2_ in O_2_, BOC gases, Geelong, VIC, USA), was thermostatically maintained at either control temperature 25 °C or heat-stress temperature 43 °C. Within the organ bath, the distal tendon of the EDL muscle was tied with braided 6/0 surgical silk (Pearsalls Ltd., Taunton, England) to a fixed pin and the proximal tendon was similarly attached to the lever arm of a dual-mode force transducer (300-CLR, Aurora Scientific, Toronto, ON, Canada). Following attachment, the EDL muscles were aligned so that platinum electrodes were equal distances on either side of the muscle. Electrical stimulus parameters, stimuli, and contractile responses were controlled, delivered, and measured using Dynamic Muscle Control and Analysis Software (DMC v5.4, Aurora Scientific, Toronto, ON, Canada).

To establish optimal muscle force production, optimal muscle length was determined at 25 °C prior to the heat-stress protocol. Optimal muscle length is defined as the muscle length that produces the maximum relative twitch tension. To establish optimal muscle length, a series of 1 Hz twitch contractions were elicited followed by small increments in muscle length, to establish peak tension [76,79]. Once optimal length was confirmed, each EDL muscle received a series of four 100 Hz stimulating pulses, 2 min apart, to assess the initial tetanic force production at 25 °C. EDL muscles allocated to group 1 (CON25) remained unstimulated for 15 min. For EDL muscles allocated to groups 2 (CON43), 3 (FVF43), and 4 (UPF43), the temperature of the organ bath was increased to 43 °C followed by the same 15 min equilibration period allowing EDL muscle to adapt to the new temperature. In all experimental groups, following the equilibration period, 100 Hz stimulations were applied every 5 min for a total of 20 min to investigate the muscle’s response to repetitive high-frequency stimulations at either 25 °C or 43 °C. The stimulating parameters used to elicit maximum tetanic contraction (Po) were based on the guidelines outlined previously [4]. At the end of the contractile protocol, EDL muscles were removed from the organ bath, weighed, and stored at −80 °C for later gene transcription analysis.

### 4.4. Gene Transcription Analysis—Real-Time Quantitative PCR (qRT-PCR)

To determine if fucoidan impacted the expression of genes known to be influenced by skeletal muscle heat stress or involved in adaptation of skeletal muscle to heat stress, quantitative real-time polymerase chain reaction (qRT-PCR) was performed on EDL muscles following the contraction protocol. RNA was extracted from EDL muscles using the Aurum™ Total RNA Fatty and Fibrous Tissue Kit (Bio-Rad Laboratories Inc., Hercules, CA, USA), then transcribed into cDNA using the iScript cDNA synthesis kit (Bio-Rad Laboratories Inc., Hercules, CA, USA). qRT-PCR was performed using an iCycler Thermal Cycler (Bio-Rad, Laboratories Inc., Hercules, CA, USA) with SsoFast EvaGreen (Bio-Rad Laboratories Inc., Hercules, CA, USA) to assess expression levels of target genes (Table 2). Expression levels then were normalized relative to the internal control gene, β-2 microglobulin (β2m), and expressed as 2^−(ΔCT)^.

### 4.5. C2C12 Tissue Culture—Temperature-Induced ROS Assay

The antioxidant capacity of fucoidan in skeletal muscle cells was determined by utilizing a heat-induced ROS production protocol in C2C12 myoblast [39,40]. Initially, a C2C12 myoblast cell line was cultured in Dulbecco’s modified Eagle’s medium (DMEM) supplemented with 10% fetal bovine serum. Cells were stored at 37 °C in a 5% CO_2_ humidified incubator and, every two days, cells were cultured to ensure confluency was maintained below 60% to prevent differentiation from occurring. Fucoidan from Fucus vesiculosus (FVF) and Undaria pinnatifida (UPF) were dissolved in Hank’s balanced salt solution (HBSS, Thermofisher, Waltham, MA, USA), then diluted to a final concentration of 500 µg/mL using DMEM supplemented with 10% fetal bovine serum. Cells were treated with either FVF, UPF, or control (HBSS) for 1 h prior to heat exposure, and maintained in the medium during the heat-stress protocol. Cells were further divided into two groups where they either remained under standard conditions (37 °C) or were placed under heat stress (43 °C). Cells allocated to the heat-stress group were incubated at 43 °C for 2 h, whilst those in the non-heat-stress group remained at 37 °C. Following the 2 h incubation, ROS levels were measured using the fluorescent probe, 2′,7′-dichlorodihydrofluorescein diacetate (H2DCFDA, D6883, Sigma Aldrich, Burlington, MA, USA). In the presence of ROS, H_2_DCFDA is oxidized to form 2′,7′-dichlorofluorescein (DCF), which is highly fluorescent at 535 nm. As per manufacturer’s instructions, C2C12 myoblasts were incubated in the dark at 37 °C in the presence of H_2_DCFDA in HBSS for 30 min, then rinsed twice with HBSS. Fluorescence was measured at 535 nm using a plate reader (Triad Multi Mode Microplate Reader, Dynex Technologies, Chantilly, VA, USA) and expressed in arbitrary units (AU).

### 4.6. Statistical Analyses

All results are presented as mean ± SEM. The statistical analyses were performed to test for differences between control (CON25), heat stress without fucoidan (CON43), and heat stress with either FVF or UPF (FVF43 and UPF43, respectively). Experimental data were analyzed using either an independent *t*-test, one-way ANOVA, or two-way ANOVA, with factors being temperature and fucoidan treatment. Where there was a significant interaction effect, a Šidāk multiple-comparisons post hoc analysis was performed for pairwise comparisons between group means. All statistical analyses were conducted using GraphPad Prism v9.1.0 (GraphPad, Boston, MA, USA), with statistical significance set at *p* < 0.05.

## 5. Conclusions

The findings from this study indicate that fucoidan extracted from Fucus vesiculosus has a protective effect against heat-induced muscle dysfunction by attenuating the loss of force, potentially through the upregulation of HSP90 expression. This highlights the potential use of fucoidan as a protective agent against heat-induced skeletal muscle dysfunction. Furthermore, our investigation demonstrates the strong antioxidant properties of fucoidan extracted from Undaria pinnatifida and Fucus vesiculosus, suggesting their potential to mitigate ROS-mediated oxidative stress, which plays a significant role in the development and progression of various diseases and conditions. Incorporating fucoidan as an antioxidant therapy could provide a novel approach to counteracting the damage to muscle tissues and promoting overall health.

## Figures and Tables

**Table 1:** Absolute mass percentages of components of fucoidan extract from Fucus vesiculosus (FVF) and Undaria pinnatifida (UPF).

Fucoidan Extract	Neutral Carbohydrates (%)	Sulfate (%)	Fucoidan (%)	Polyphenols (%)	Peak Molecular Weight (kDa)
FVF	62.7	25	92.9	3.3	49.6
UPF	43.5	25.9	86	<2	46.8
Carbohydrate breakdown (mass %) of neutral carbohydrates					
**Fucoidan Extract**	**Fucose (%)**	**Xylose (%)**	**Galactose (%)**	**Arabinose (%)**	**Rhamnose (%)**
FVF	46	7	4	1	0
UPF	21	1	18	1	0

**Table 2:** Mouse primers for quantitative RT-PCR.

	Forward Primer (5′ to 3′)	Reverse Primer (5′ to 3′)
β2M	GTATGCTATCCAGAAAACCC	CTGAAGGACATATCTGACATC
*HSF1*	AGAGAAAGATCCCTCTGATG	AGTGATATCGGAGATTTATGGG
*NRF2*	CTAGCCTTTTCTCCGCCTTT	GAGGCTACTTGCAGCAGAGG
*HSP27*	CTTCACCCGGAAATACAC	CGAAAGTAACCGGAATGG
*HSP70*	AGTTCTTTGTGTTTGGACTC	TAACAGTCAACGCAATTACC
*HSP90*	GCGGCAAAGACAAGAAAAG	CAAGTGGTCCTCCCAGTCAT

## Data Availability

The data presented in this study are available at https://figshare.com/s/ffd6f2ca18def259114b (accessed on 21 November 2023).

## Abbreviations

The following abbreviations are used in this manuscript:

ANGPT	Angiopoietins
ANOVA	Analysis of variance
CCL2	C-C motif ligand 2
°C	Degrees celsius
EDL	Extensor digitorum longus
EMG	Electromyography
FOXO	Forkhead box family
FVF	Fucus vesiculosus
H2DCFDA	2′,7′-dichlorofluorescein
HBSS	Hanks’ balanced salt solution
HMWF	High-molecular-weight fucoidan
HSF	Heat shock factor
HSP	Heat shock protein
LMWF	Low-molecular-weight fucoidan
MMWF	Medium-molecular-weight fucoidan
MRT	Mean residence time
NAC	N-acetylcysteine
NRF	Nuclear factor erythroid 2-related fact
qRT-PCR	Real-time quantitative reverse-transcription PCR
ROS	Reactive oxygen species
SIRT	Sirtuin
O_2^−^_	Superoxide
UPF	Undaria pinnatifida
VEGF	Vascular endothelial growth factor
